# A lightweight St-CNN architecture based on deep learning for stress level detection from human physical activities

**DOI:** 10.1038/s41598-025-18647-x

**Published:** 2025-09-29

**Authors:** İsmail Kayadibi, Osman Uslu

**Affiliations:** Department of Management Information Systems, Faculty of Economic and Administrative Sciences, Afyon Kocatepe University, Afyonkarahisar, 03200 Turkey

**Keywords:** Artificial intelligence, Convolutional neural network, Stress detection, Edge computing, Biomedical engineering, Psychiatric disorders

## Abstract

Stress significantly affects both daily and professional life, highlighting the need for effective management strategies and continuous monitoring. While many methods have been developed, achieving accurate and objective stress detection remains a key challenge. This study proposes a lightweight Stress Convolutional Neural Network (St-CNN) architecture designed to detect individual stress levels based on physical activity data. The method was evaluated using the publicly available Stress-Lysis dataset, which contains 2,001 samples with features such as body temperature, humidity, and step count. The St-CNN model is composed of two fully connected layers, along with ReLU and normalization layers, forming a streamlined architecture optimized for low computational cost. It achieved a perfect accuracy rate of 100% and outperformed traditional machine learning (ML) methods, including Decision Tree (DT), K-Nearest Neighbors (KNN), and Support Vector Machine (SVM). The model was additionally validated through 10-fold cross-validation with 95% confidence intervals, achieving an accuracy of 99.85%. The proposed method outperformed state-of-the-art approaches on the Stress-Lysis dataset, achieving superior performance with a lightweight architecture for stress level detection. In conclusion, the proposed St-CNN architecture provides a practical and efficient approach for real-time stress monitoring in edge computing environments, combining high classification accuracy with minimal computational overhead.

## Introduction

Stress is defined as a physiological and psychological response to external or internal stimuli that disrupt an individual’s equilibrium. While short-term stress may enhance alertness and performance under certain circumstances, prolonged or recurrent exposure is widely recognized to have detrimental effects on physical and psychological well-being, particularly in occupational and social contexts^1– 2^. The severity of these effects is shaped by the nature of the stressor, individual susceptibility, and the frequency and duration of exposure, and stress is generally categorized into acute, episodic acute, and chronic types^3–5^. Acute stress is typically transient and may even exert performance-enhancing effects^6–8^, whereas episodic acute and chronic stress are associated with sustained physiological and emotional burden, contributing to conditions such as depression, burnout, cardiovascular complications, and immunosuppression^9–14^. These adverse consequences are also well documented in organizational settings, where stress has been linked to absenteeism, decreased productivity, low motivation, and workplace disengagement^2,15^.

In response to the widespread implications of unmanaged stress, various institutions—ranging from healthcare providers to academic and corporate organizations—have developed intervention strategies aimed at early detection and effective management^16–18^. A fundamental prerequisite for such interventions is the ability to accurately and objectively assess stress levels. Although traditional assessment methods such as biochemical sampling, self-report questionnaires, psychometric scales, and behavioral observation are still widely employed^19–21^, these approaches are often limited by subjectivity, delayed feedback, and lack of scalability. In contrast, physiological indicators such as heart rate, skin conductance, respiratory rate, muscle tension, and body temperature provide real-time, quantifiable markers of stress^22^. Empirical evidence has consistently demonstrated the efficacy of such physiological parameters—particularly electrodermal activity, electromyography, and heart rate variability—in capturing stress-related changes with greater precision and reliability^23–25^. Nonetheless, the routine applicability of these techniques remains constrained by operational limitations, including cost, portability, and the need for specialized equipment.

Recent advancements in artificial intelligence (AI) and the Internet of Things (IoT) have catalyzed the development of wearable systems capable of monitoring physiological signals in real time and in everyday environments^26–28^. These innovations offer a promising direction for continuous, non-invasive stress detection that is not only scalable and cost-effective but also suitable for integration into daily life. Beyond individual health benefits, such systems contribute to improved workplace efficiency, early intervention in stress-related conditions, and broader public health outcomes, thereby supporting the long-term sustainability of healthcare systems and workforce productivity^29–34^. Accordingly, the objective classification of stress levels through lightweight AI-driven architectures integrated with physiological data collection represents a critical research trajectory with significant practical and theoretical implications.

AI has recently revolutionized healthcare by demonstrating effective performance in various medical processes, including early detection and diagnosis, treatment, and outcome prediction^35^. Deep learning (DL), a subfield of AI, has shown remarkable success in uncovering complex structures in tasks such as image recognition and speech recognition^36^. Moreover, the integration of the IoT with AI methods like DL has led to significant advancements in daily life and healthcare. The combination of wearable sensors or devices with IoT and AI techniques allows healthcare professionals to diagnose and intervene in patients’ conditions at an early stage, thereby reducing potential risks^37^. IoT devices have become particularly prominent in medical applications based on wearable sensors due to their numerous benefits, including electronic information management, controlled communication and data processing, ease of use, low cost, and enhanced patient satisfaction^38^. In recent years, the use of DL methods—particularly advanced techniques such as convolutional neural networks (CNNs)—has become increasingly widespread, especially in domains such as healthcare, due to their effectiveness in identifying complex patterns within large-scale datasets^39^. In this context, large datasets obtained from wearable sensors that provide real-time data via IoT can be efficiently processed using DL techniques, allowing for the simultaneous examination of various attributes to generate patterns. A key reason for the success of DL tools is their ability to automatically build feature hierarchies and extract generalized trends from complex big data^36^. In addition, the broader subset of IoT that focuses on healthcare applications, such as patient monitoring and data sharing, is known as the Internet of Medical Things (IoMT). IoMT aims to provide more efficient, data-driven solutions in patient care and healthcare by leveraging the connectivity and data analysis capabilities offered by IoT^40^. IoMT devices typically include wearable health sensors, smart medical devices, patient monitoring systems, and similar technologies. These devices continuously collect medical data, making the rapid analysis of this data critically important. Consequently, the use of AI technologies in specialized fields such as biomedicine to analyze the data collected by IoMT devices has recently become more prevalent^41^. While single-use IoMT devices are generally designed to transmit real-time data to cloud databases, edge computing-based IoMT approaches focus on processing data at or near the source of data generation, rather than relying on centralized data processing warehouses^42^. In the context of healthcare, edge computing involves processing health data at or near the medical devices where it is generated, such as sensors and wearables. This approach offers several advantages, including lower latency, enhanced data privacy and security, and improved scalability and reliability^42^. Moreover, one of the most significant benefits of edge computing is its ability to enable real-time data processing while maintaining data privacy and security. As a result, the edge computing AI approach, which combines edge computing and AI for healthcare applications, has recently gained popularity and is becoming increasingly widespread^43^.

Stress, recognized as a common health issue that causes various negative effects on humans, can be effectively identified and managed through continuous monitoring and analysis of physiological signals using IoT-based wearable sensors^44^. With the advancement of technology, recent progress in IoT and DL has significantly enhanced the monitoring and classification of physiological parameters related to human activity, particularly in the context of stress. For example, wearable sensor methodologies like Stress-Lysis have been developed to detect and monitor stress by measuring human activity-related physiological parameters, such as body humidity and temperature^45^. By leveraging the effective data processing capabilities of multilayer artificial neural networks, DL can more accurately and efficiently detect and manage stress by processing complex data from wearable sensors. Additionally, the integration of DL algorithms into IoMT devices has facilitated the analysis of wearable sensor data, enabling customized feedback and interventions to help users manage stress. The use of DL and IoMT for stress detection also holds the potential to increase efficiency and accuracy compared to traditional classification methods. DL enables real-time analysis of IoMT-acquired data, allowing for the early detection of stress-related conditions that could negatively impact quality of life. In summary, the application of IoT devices such as IoMT and DL in continuous physiological signal monitoring, personalized treatment planning, and feedback mechanisms underscores its transformative potential in the healthcare sector.

Our research aims to develop both practical applications and theoretical frameworks by analysing stress data from wearable sensors through IoMT for the classification of stress levels. In particular, we propose a Stress Convolutional Neural Network (St-CNN) architecture designed to detect stress levels based on physical activity data. This work makes several important contributions. First, building on existing research on physiological stress level determination, our methodology improves sensitivity and reliability by categorising stress levels as low, moderate and high. This progress facilitates the implementation of targeted interventions and enables more accurate predictions. Second, we extend research and deepen understanding in the field of stress categorisation. Finally, our proposed model provides an accurate and practical tool that can be integrated into various devices for real-world applications by leveraging IoMT-based edge computing AI systems. The performance of the proposed architecture was evaluated using the widely recognised Stress-Lysis dataset and the findings were reported. In summary, based on the proposed methodology and its significant contributions to the literature, the main objectives of this work are as follows:

1. Proposing a DL-based St-CNN architecture with lightweight and robust performance for stress level detection,
2. Comparison of the performance measures of the proposed method on the Stress-Lysis dataset widely used in the literature,
3. Presenting a performance comparison of the proposed method with ML methods such as SVM, KNN and DT,
4. Performing the optimal partition ratio research with the proposed method for the Stress-Lysis dataset,
5. Evaluation of the proposed method on the Stress-Lysis dataset and comparison of the findings with state-of-the-art approaches,
6. To contribute to the literature by proposing a superior and highly effective state-of-the-art St-CNN architecture for edge computing-based AI applications in stress level detection, improving upon previous studies.

The rest of the paper is structured as follows: Sect. 2 contains a detailed literature review on the topic of this work. Section 3 presents an overview of the proposed method, the Stress-Lysis dataset, the CNN, the proposed lightweight St-CNN architecture, and a detailed description of the ML techniques used. Section 4 details the experiments conducted to evaluate the performance of the proposed method and the results obtained. Section 5 discusses the main contributions of this work compared to state-of-the-art technologies, using comparative performance analyses of the findings. Finally, Sect. 6 provides a concluding section summarizing the study’s findings.

## Related works

Various AI-based ML and DL approaches have recently been developed in the literature to utilize physiological parameters related to human activity. In this context, approaches similar to those in this study, particularly those based on the Stress-Lysis dataset, are analyzed in detail, and a literature review on the subject is presented below.

In one of the AI studies based on human activity, Mohod and Jawandiya (2024) developed a SVM algorithm for stress detection using human activity-related parameters, validating their algorithm on the Stress-Lysis dataset and achieving 99% accuracy^46^. Al-Atawi et al. (2023) introduced an ML-based system, Stress-Track, designed to monitor an individual’s stress levels during physical activity. The developed system reportedly contributes to improved stress management and healthcare services, achieving an impressive accuracy rate of 99.5%^38^. Rachakonda et al. (2019) proposed a novel DL-based system, known as the Stress-Lysis system, to detect stress levels during physical activity. The system was trained and tested on a dataset comprising physical activity parameters such as body temperature, step count, and body humidity. The test results indicated that the proposed method achieved a high accuracy of 99.7%^45^. Suraj Arya and Ramli (2024) used various ML algorithms and DL methods, including Multi-Layer Perceptron and Artificial Neural Networks, to detect stress at an early stage. They evaluated the performance of these methods on a stress dataset collected from students at Dharan University in Nepal, with the Naive Bayes, a ML model, achieving the highest performance with 90% accuracy^47^. Bobade and Vani (2020) proposed different ML and DL techniques for stress detection in individuals and validated the performance of their methods using a multimodal dataset recorded from wearable physiological and motion sensors. Their evaluation revealed that the ANN, a DL method, could detect stress with 95% accuracy^48^. Kadu et al. (2024) proposed an IoT-based stress detection system that utilizes physiological signals collected via wearable sensors and employs various ML algorithms, such as Random Forest and SVM, achieving an accuracy ranging from 98.6 to 99.8%^49^. Building upon the need for higher accuracy and deeper temporal feature extraction, Mukherjee and Roy (2024) introduced an attention-based CNN-TLSTM architecture that integrates electroencephalography (EEG) signals and pulse rate data. By combining spatial and temporal feature learning with an attention mechanism, their model further improved performance, achieving an impressive accuracy of 99.72% and demonstrating the potential of advanced DL methods in multimodal stress assessment^50^.

The literature review on stress detection indicates that the stimuli triggering signals received by wearable sensors from individuals are known as stress factors. These factors are collected through wearable sensors and devices and can be stored in cloud environments, supported by IoT technology, which is necessary for the functioning of cloud processing centers that rely on internet connectivity. As discussed earlier, various IoT-based AI methods have been proposed to measure stress levels in different scenarios^45–49^. Additionally, the literature highlights a range of measurement techniques for detecting stress, including biomarkers such as electrocardiography, EEG signals, skin conductance, respiration, and heart rate variability. The importance of developing effective solutions for stress detection has become increasingly clear, given the rising global awareness of stress-related issues. Most researchers, as noted in the literature, have focused on detecting stress through human activity parameters, leading to the recent development of various DL and ML methods. Also, Al-Atawi et al.^38^ have pointed out that the performance of existing methods for automatic stress detection and classification is still inadequate. Summarizing the literature, it is clear that there is still a need to develop methods that demonstrate strong performance in stress detection while maintaining a lightweight design for IoMT-driven edge computing AI approaches. To address these gaps in the literature, this study proposes a DL-based St-CNN architecture aimed at delivering strong classification performance with lightweight design. The effectiveness of the proposed method is thoroughly evaluated using the publicly available Stress-Lysis dataset, providing a meaningful benchmark for comparison within the literature. The results suggest that the proposed method could be a valuable tool for IoMT devices used in stress measurement, demonstrating notable and reliable classification performance. Lastly, the findings obtained with proposed method are compared with state-of-the-art approaches validated with this dataset, and a detailed performance comparison analysis is presented in the discussion section.

## Materials and methods

In light of the insights gained from the literature review, this paper aims to achieve robust performance with a lightweight DL architecture for stress detection in wearable-based edge computing devices. To this end, a CNN method, recognized as a powerful DL method, is employed, and a novel approach is proposed. All relevant details regarding the development of the proposed method are presented in this section.

### Overview of the proposed method

**Fig. 1:**
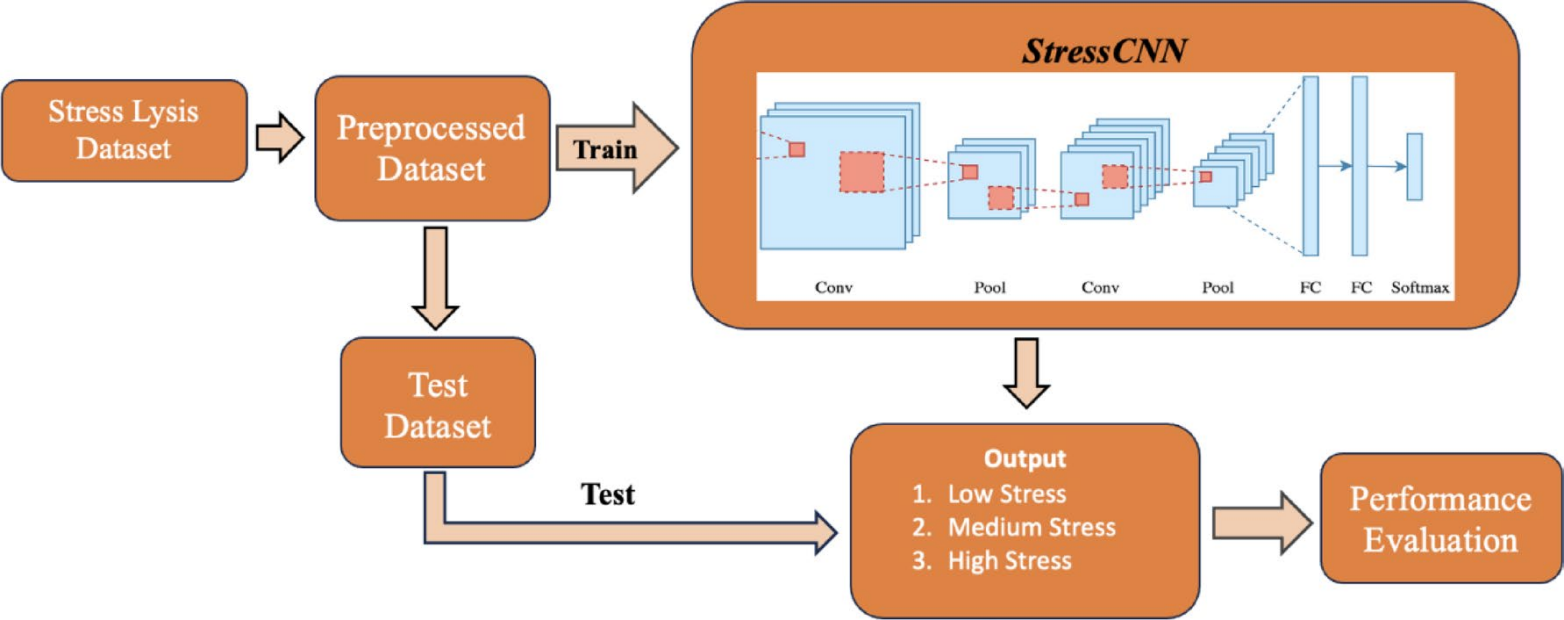
Flow diagram of proposed lightweight St-CNN architecture.

In this study, we proposes a lightweight St-CNN architecture for stress detection based on an individual’s physical activity, utilizing stress parameters collected from wearable devices via IoMT. The performance of the proposed architecture is evaluated through a comparative analysis with common ML methods. Furthermore, a flow diagram of the proposed St-CNN architecture is also presented in Fig. 1.

As depicted in Fig. 1, the proposed methodology is comprised of five distinct steps. Initially, a dataset encompassing parameters related to human activity, such as body humidity and temperature, was examined for relevance to the proposed method. The Stress-Lysis dataset, a recent addition to the literature and frequently utilized for this purpose, was selected. This dataset, which contains numerical data, was checked for missing and incorrect entries; however, no significant missing data or extreme outliers were identified. To ensure consistency in feature scales, z-score normalization was applied to standardize the input features. As a result, a pre-processed dataset was obtained by using stress-related parameters as feature inputs and stress level as categorical labels before being included in the St-CNN architecture. In the subsequent step, the preprocessed dataset was utilized to train the St-CNN model, following the optimization of hyperparameters. Upon completion of the training phase, the model was evaluated using the test subset of the dataset. The efficacy of the proposed method was then assessed based on the performance metrics established in this study. Lastly, a comparative analysis was conducted between the performance of the proposed method and several established ML techniques, including DT, SVM, and KNN, as documented in the literature. This comparison, along with the detailed results and performance metrics of the proposed method, is presented and contrasted with state-of-the-art methods. The subsections of this section provide a comprehensive account of all steps and processes involved in the development of the proposed method.

### Stress-lysis dataset

**Fig. 2:**
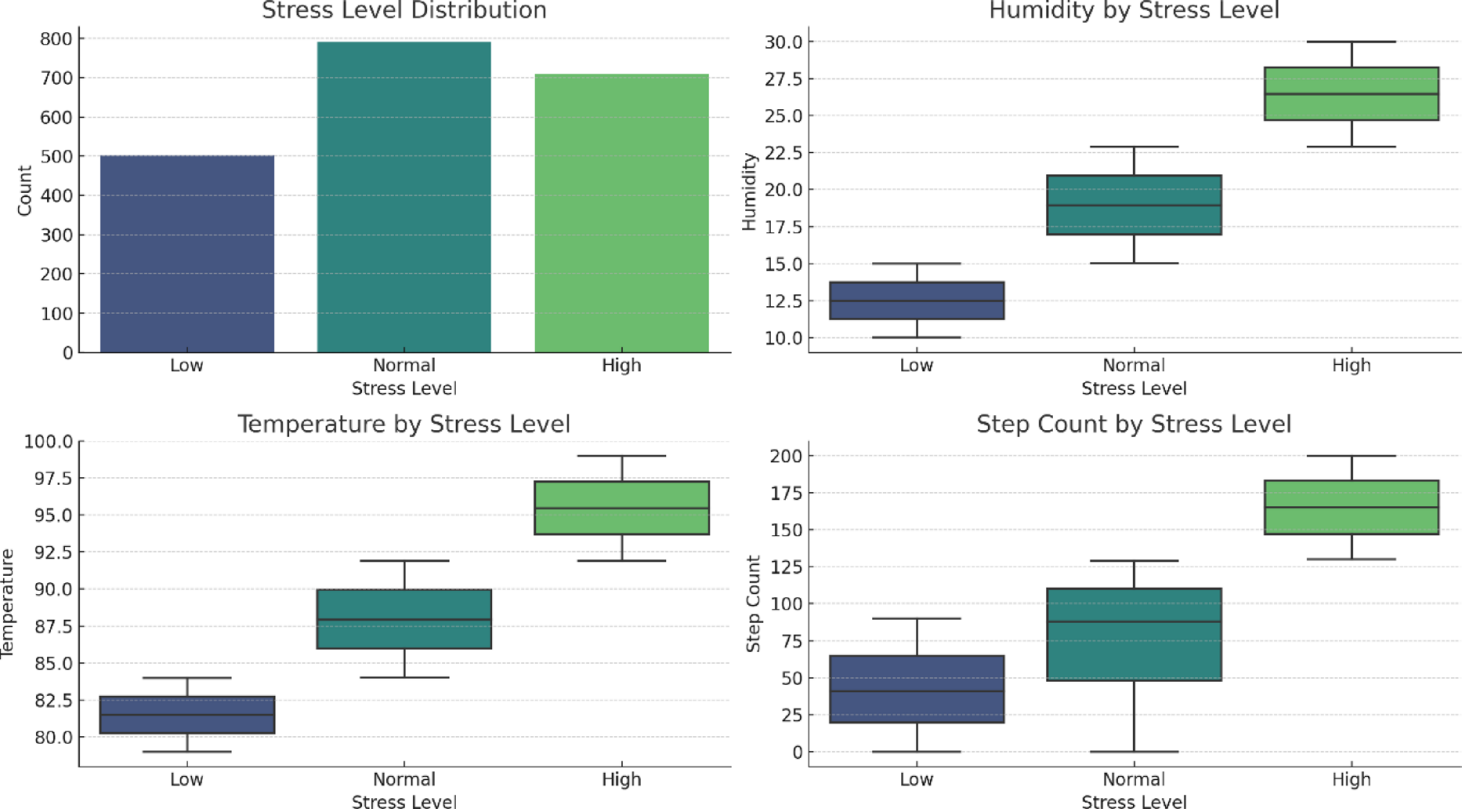
Details of stress-lysis dataset according to stress level classes.

Human stress detection has recently garnered significant attention, particularly within the domains of wearable technology and healthcare, owing to advancements in technology. In this study, we utilize the Stress-Lysis dataset, which is comprised of physiological metrics such as body moisture, body temperature, and step count, collected from wearable devices to assess stress levels based on physical activity. This dataset includes 2,001 samples of physiological data, categorized into three distinct stress levels—low stress (501 samples), normal stress (790 samples), and high stress (710 samples)—and is publicly accessible on Kaggle for research purposes^45^. The characteristics of the Stress-Lysis dataset and its distribution according to stress categories are shown in Fig. 2.

A notable limitation of this dataset is its lack of pre-defined training and test sets for classification tasks. Therefore, in this study, the dataset was randomly partitioned into 80% for training and 20% for testing the proposed method. Additionally, performance evaluations were conducted using various random data splitting ratios to identify the optimal configuration that maximizes classification accuracy. The ratio yielding the highest performance was subsequently selected for proposed method. Additionally, in this study, a comprehensive performance evaluation was carried out through cross-validation, and the corresponding 95% confidence intervals were established to assess the reliability of the results. All findings obtained from these evaluations have been presented in detail.

### Convolutional neural network (CNN)

**Fig. 3:**
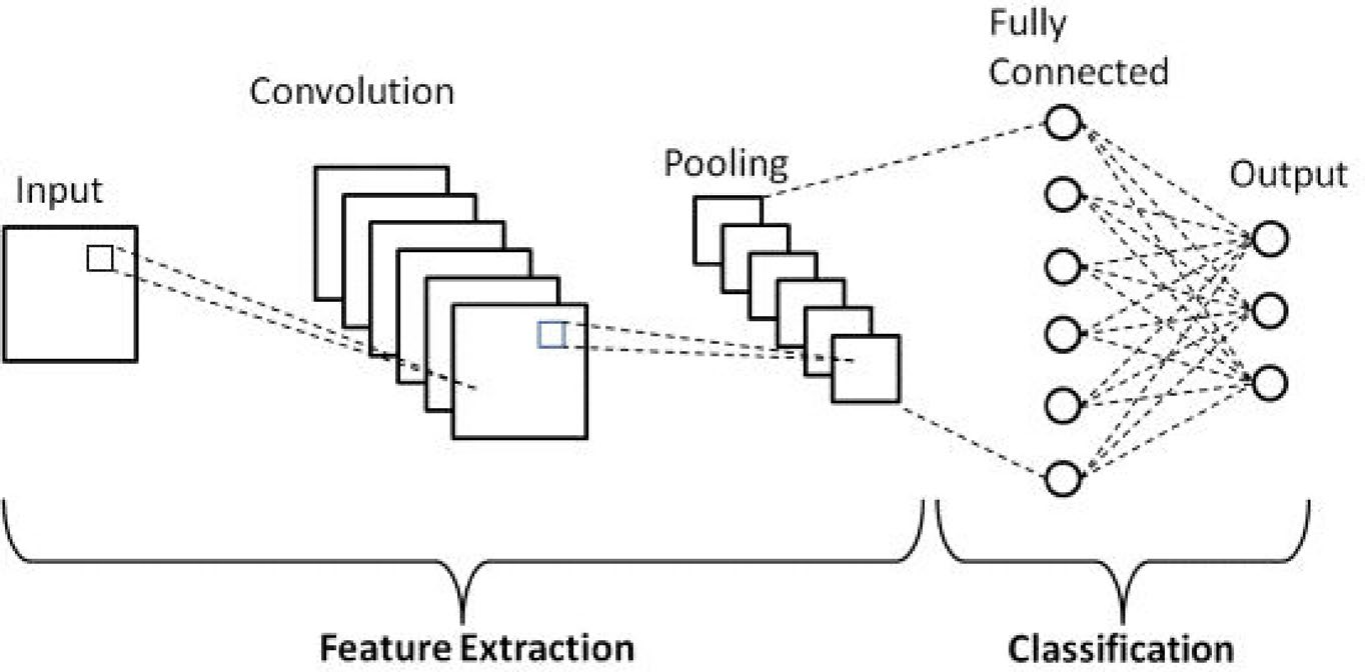
Basic structure of a CNN architecture.

DL represents a specialized domain within ML that employs multilayer neural networks to model complex data representations^36^. As an AI methodology, DL is designed to enable computers to process and interpret data in a manner analogous to human cognitive processes. Recent advancements in DL have led to significant breakthroughs in addressing problems related to speech recognition, natural language processing, and image analysis, owing to its capacity to analyze and extract features from complex structures within extensive datasets, supported by progress in technological infrastructure and computational capabilities^36^. A prominent DL technique, CNN is distinguished by its efficacy in feature extraction, facilitated by its hierarchical architecture. CNNs, which derive their name from the convolution operation, incorporate convolutional layers followed by pooling and fully connected layers to systematically extract and classify features from data. The architecture of a fundamental CNN model is depicted in Fig. 3.

As depicted in Fig. 3, a fundamental CNN architecture comprises several key layers: input, convolutional, pooling, fully connected, and output layers. The convolutional layers, which are central to the CNN’s functionality, are responsible for feature extraction from the input data. These layers are followed by the pooling layer, which reduces the dimensionality of the convolutional feature maps, thereby decreasing computational requirements and mitigating overfitting. Subsequent to the pooling layer, the Fully Connected (FC) layer is employed, wherein neurons are interconnected with weights and biases across different layers. This layer facilitates the integration of features extracted by the convolutional and pooling layers, leading to the initiation of the classification process and culminating in data classification. A pivotal element in CNN models is the activation function, which is instrumental in capturing complex, non-linear relationships between variables within the network. Common activation functions, such as the Rectified Linear Unit (ReLU), are typically applied following the convolutional and pooling layers^36^. These functions enhance the network’s ability to learn and represent intricate data patterns, thereby improving accuracy in classification and object recognition tasks. In summary, each layer in a CNN architecture has a distinct role and contributes to the network’s overall efficacy. The depth of a CNN, indicated by the number of convolutional layers, generally correlates with its ability to model complex data. However, optimal performance may not always necessitate deep architectures. Therefore, the depth of a CNN should be calibrated to achieve satisfactory performance for specific applications. In this study, CNN, one of the DL techniques for stress detection, is chosen due to its unique ability to efficiently process and learn features from raw data, especially images. Unlike traditional ML methods that require manually crafted feature extraction by domain experts, CNNs automatically discover complex patterns at multiple levels of abstraction. This makes them particularly effective in tasks such as visual recognition and image classification^36^. The CNN model developed in this study is meticulously designed to achieve high performance through a relatively lightweight architecture optimized for edge computing applications. This approach is particularly advantageous for wearable devices leveraging IoT technology, which are often constrained by limited computational resources, thereby necessitating efficient and resource-aware solutions.

#### Proposed lightweight St-CNN architecture

**Fig. 4:**
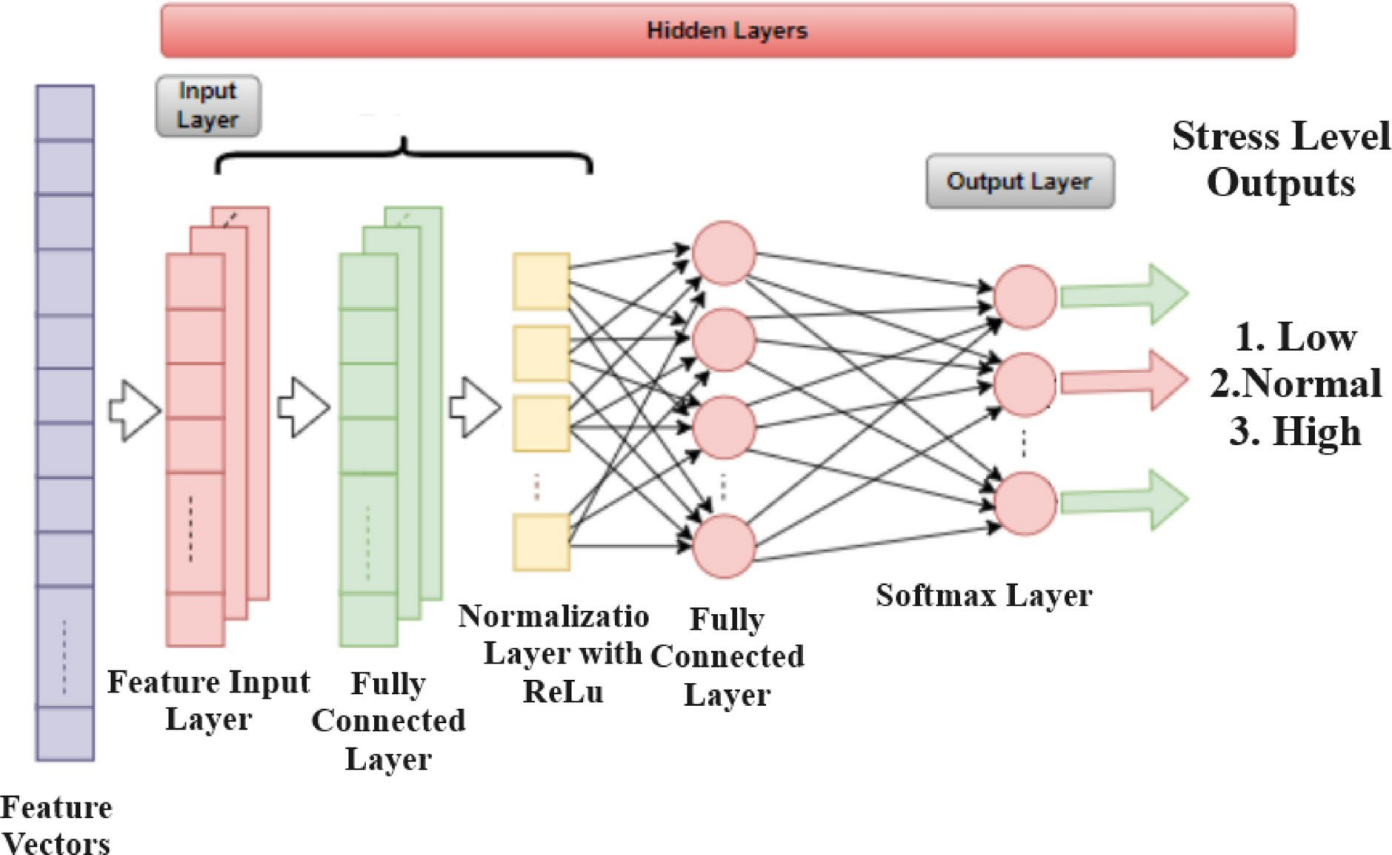
Proposed lightweight St-CNN architecture.

In this study, we propose a lightweight St-CNN architecture that offers rapid operational flexibility for detecting stress levels based on human activities. The structure of this proposed architecture is illustrated in Fig. 4.

As illustrated in Fig. 4, the proposed lightweight St-CNN architecture is composed of five primary layers: a feature input layer, two fully connected (FC) layers, one layer normalization layer, a ReLU activation layer, and a softmax output layer. The input layer processes a feature vector consisting of three physiological parameters—Humidity, Temperature, and Step Count—which are standardized using z-score normalization to enhance numerical stability and convergence. The first fully connected layer maps the 3-dimensional input to 16 hidden units. This is followed by a layer normalization operation and a ReLU activation function to introduce non-linearity and stabilize internal representations. The second fully connected layer maps the 16-dimensional hidden representation to three output neurons corresponding to discrete stress level classifications (e.g., low, moderate, high). A softmax layer is then applied to convert raw scores into class probabilities.

Unlike CNNs, the St-CNN does not include any convolutional or pooling layers; thus, configuration parameters such as kernel size or stride are not applicable. Instead, the architecture adopts a fully connected design tailored for low-dimensional physiological data input. In terms of computational complexity, the architecture is highly efficient: it contains a total of 115 trainable parameters and requires approximately 287 floating-point operations (FLOPs) per inference. This results in an extremely compact memory footprint of just 0.46 KB (assuming 32-bit floating-point weights). Such efficiency makes the model particularly well-suited for deployment in resource-constrained environments, such as edge computing devices, wearable health monitors, and embedded systems, where real-time processing and low power consumption are critical requirements.

The effectiveness of the proposed architecture has been systematically evaluated across a range of performance scenarios. In addition to reporting high classification accuracy, the method has been benchmarked against conventional ML techniques, demonstrating superior performance with significantly lower computational cost. This combination of accuracy and efficiency reinforces the suitability of the St-CNN architecture for practical, real-world stress detection applications in ubiquitous and mobile computing environments.

### Machine learning (ML) methods

The domain of AI encompasses a broad spectrum of ML algorithms, each characterized by distinct advantages and limitations that vary with the specific context of their application. Among these algorithms, decision tree (DT), k-nearest neighbors (KNN), and support vector machines (SVM) are frequently employed in classification problems and are noted for their potential to deliver effective performance, particularly when utilized in conjunction with CNN models^51^. A DT is a classical supervised ML technique that represents decision-making processes through a tree-like structure, which visually delineates various decision paths and their associated outcomes^52^. SVM are another supervised learning technique that aims to identify the optimal hyperplane that maximizes the margin between distinct classes, thereby enhancing classification accuracy^53^. The KNN algorithm is a fundamental method in ML, commonly used for classification and regression tasks based on the proximity of data points in the feature space^54^. This method classifies data based on the majority class or the average value of the “k” nearest neighbors. In the present study, a variety of ML algorithms, including KNN, SVM, and DT, were utilized to evaluate the performance of the proposed St-CNN model. A comparative analysis of these methods was conducted to benchmark the efficacy of the St-CNN model.

## Experimental results

This study introduces the St-CNN model for the detection of human stress levels based on physiological parameters. As depicted in Fig. 1, the Stress-Lysis dataset, which includes physiological data such as body moisture, body temperature, and step count, was employed to evaluate the performance of the proposed method. Initially, the dataset was preprocessed to address any missing or erroneous data before being input into the St-CNN model. The St-CNN architecture was then trained according to predefined hyperparameters and subsequently tested on a reserved subset of the dataset. Performance metrics for the St-CNN model were computed using a confusion matrix, and the overall performance was documented. Additionally, ML algorithms, including KNN, SVM, and DT, were trained and tested on the Stress-Lysis dataset to facilitate a comparative performance analysis. Metrics for these algorithms were also calculated and reported. A performance comparison table, detailing the results of the proposed method against the traditional ML algorithms, was presented and analyzed. Finally, the St-CNN model was evaluated across various data partition ratios to identify the optimal ratio, with the most effective partition ratios being determined and applied in the proposed method. The performance metrics obtained for the St-CNN model on the Stress-Lysis dataset are presented, with comprehensive details of the experimental procedures provided in the subsequent subsections.

### Experimental setting

All experiments related to the proposed method and the ML techniques utilized in this study were performed using MATLAB 2023b within a computational environment conFig.d with the Windows 11 operating system, an Intel^®^ Core™ i7 processor operating at 2.3 GHz, and an NVIDIA GeForce RTX 4050 graphics card.

###  Hyperparameter selection

The selection of hyperparameters is a critical factor in optimizing the performance of CNNs^55^. Hyperparameters, which are parameters used to conFig. the CNN architecture, significantly influence the efficiency of the training process, the network’s generalization performance, and the mitigation of overfitting risks. Therefore, it is imperative to meticulously select hyperparameters and conduct rigorous experimentation or utilize hyperparameters with proven efficacy documented in the literature. Key hyperparameters in CNNs include the loss function, optimization algorithm, learning rate, L2 regularization, batch size, gradient decay factor, and the number of epochs^56^.

For the proposed St-CNN model, validated performance metrics reported in the literature were selected, and a series of systematic experiments were conducted to determine the optimal hyperparameters that provide the highest performance. As a result of these experiments, the hyperparameters that ensure performance consistency for the proposed method were identified and are presented in Table 1. Additionally, to ensure the robustness of the hyperparameter selection and to control the risk of overfitting, a cross-validation strategy was employed during the training of the proposed St-CNN architecture.

**Table 1:** Hyperparameters determined for the training of the St-CNN architecture.

Parameter	Value/type
Loss criterion	Crossentropyex
Optimizer	Adam
Learning rate	0.001
L2 Regularization	0.0001
Batch size	128
GradientDecayFactor	0.9
Epoch	200
Network output	3 (Number of classes)
Cross Validation	10-fold

In this study, as illustrated in Fig. 1, the proposed method was trained on the designated training portion of the Stress-Lysis dataset, employing the hyperparameters detailed in Table 1. As depicted in Fig. 4, the St-CNN architecture was engineered to automatically extract hierarchical features from the data via its input layer, fully connected layers, normalization layers, and the ReLU activation function. The final output layer, utilizing a softmax activation function, enables the model to produce probabilistic predictions of stress levels. The model’s prediction error, defined as the deviation between the predicted outputs and the actual labels, is computed to guide the training process. To minimize this error, weight updates are performed using the backpropagation algorithm, thereby facilitating the model’s learning. The lightweight configuration of the St-CNN architecture contributes to expedited training processes. During training, cross-entropy loss was employed as the loss criterion to assess the accuracy of the model’s predictions against the true labels. Also, the loss criterion is the categorical cross-entropy loss (also known as softmax loss). The mathematical expressions used for cross-entropy and its loss function are given Eqs. 1 and 2. Following used the loss function, the softmax function was used to convert the model’s output into a probability distribution over the class labels. The mathematical formulation for softmax function are provided in Eq. 3.

1 $$\:H\left(p,q\right)=\:-\sum\:_{x}p\left(x\right)\text{log}q\left(x\right)$$

2 $$L = - \frac{1}{N}\mathop \sum \limits_{{i = 1}}^{N} \mathop \sum \limits_{{c = 1}}^{C} y_{{i,c}} \log \left( {p_{{i,c}} } \right)$$

3 $$\:softmax{\left(z\right)}_{i}=\:\frac{{e}^{{z}_{i}}}{{\sum\:}_{j=1}^{K}{e}^{{z}_{j}}}$$

In Eq. 1, $\:x$ represents the number of classes, $\:q$ denotes the output of the softmax function, and $\:p$ represents the categorical class output. In Eq. 2, $\:N$ is the number of training examples (batch size), C is the number of classes, $\:{y}_{i,c}$​ is a binary indicator (0 or 1), and $\:{p}_{i,c}$​ is the predicted probability for class $\:c$. In Eq. 3, $\:softmax{\left(z\right)}_{i}$ refers to the softmax transformation of the i-th element of the vector $\:z$. Additionally, $\:i$ represents the index of the element, $\:e$ denotes Euler’s number, and $\:k$ represents the dimensionality of the vector $\:z$.

**Fig. 5:**
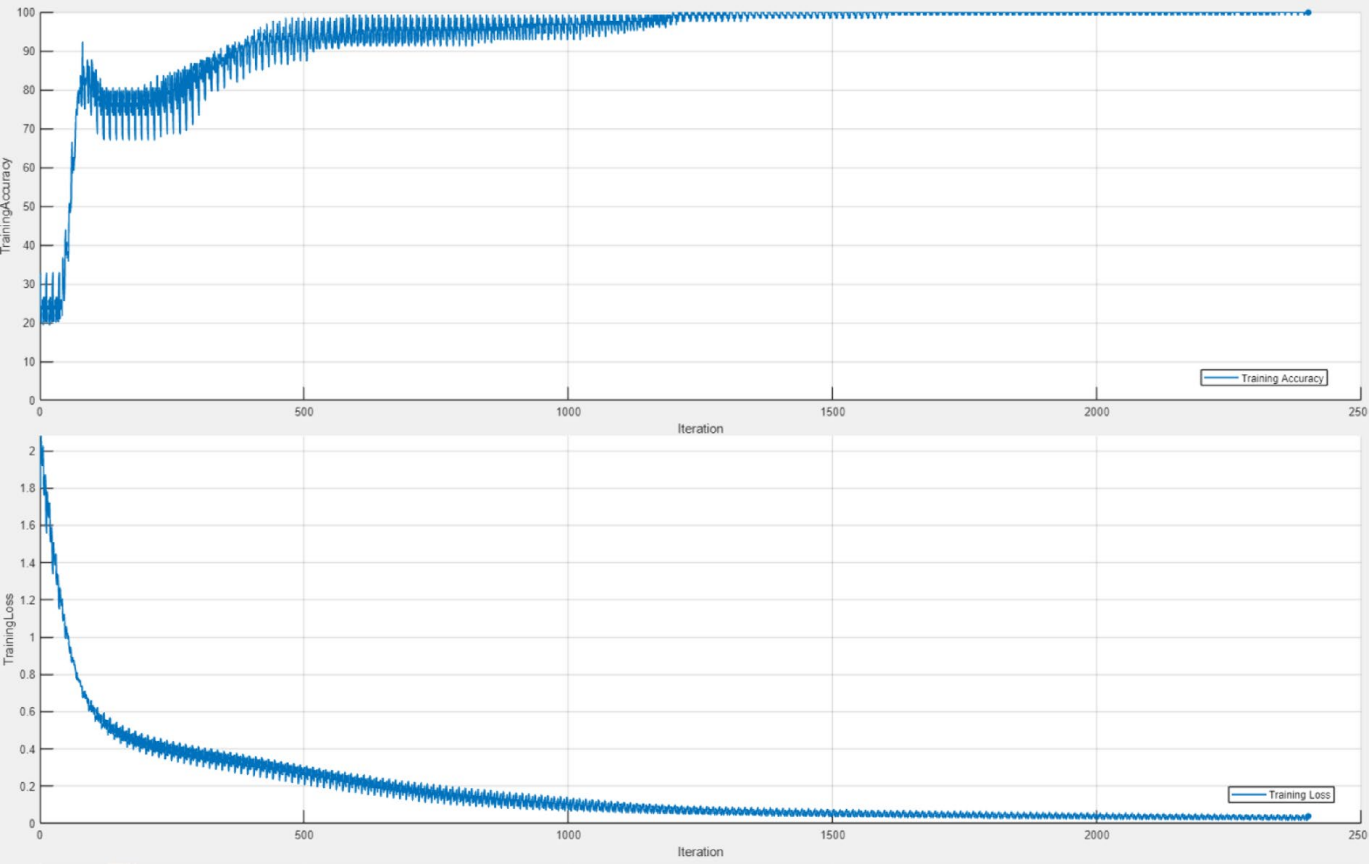
Training accuracy vs. loss graphs of the St-CNN Architecture.

The model was trained for 200 epochs, a number chosen based on experimental observations to achieve optimal convergence without significant overfitting. Throughout this training period, the model’s parameters were iteratively refined, and its generalization performance was improved using the Adam optimizer. The Adam optimizer is a widely used optimization algorithm that consistently achieves superior performance in DL methods compared to other optimization techniques such as SGDM and RMSProp^56^, and is renowned for its robust convergence properties achieved through adaptive updates of network parameters^57^. Following the completion of the training phase, the accuracy and loss curves generated during the training process are depicted in Fig. 5. These curves correspond specifically to the training of the St-CNN model using a 80% training and 20% testing split of the dataset, which achieved one of the highest performance results among the evaluated configurations. It should be noted, however, that the overall performance of the proposed method was also comprehensively assessed through additional evaluations, including 10-fold cross-validation and various data splitting strategies.

The training accuracy and loss curves shown in Fig. 5 exhibit a stable convergence behavior, with accuracy nearing 100% and loss approaching zero, without indications of overfitting such as oscillations or divergence. These results confirm the effectiveness and generalization capability of the proposed St-CNN architecture.

### Performance metrics

In this study, the performance of the St-CNN architecture was assessed using a confusion matrix, a fundamental tool in classification tasks^58^. The confusion matrix facilitates the computation of several key performance metrics, such as accuracy, sensitivity, specificity, precision, and the F1 score. It provides a detailed overview of how well the model’s predictions align with the actual class labels and is composed of four principal components:

True Positives (TP): TP indicates correct stress detection for individuals experiencing stress.

True Negatives (TN): TN indicates correct identification of individuals who are not experiencing stress.

False Positives (FP): FP indicates when the model incorrectly predicts stress for individuals who are not experiencing stress.

False Negatives (FN): FN indicates when the model fails to detect stress in individuals who are experiencing stress.

**Fig. 6:**
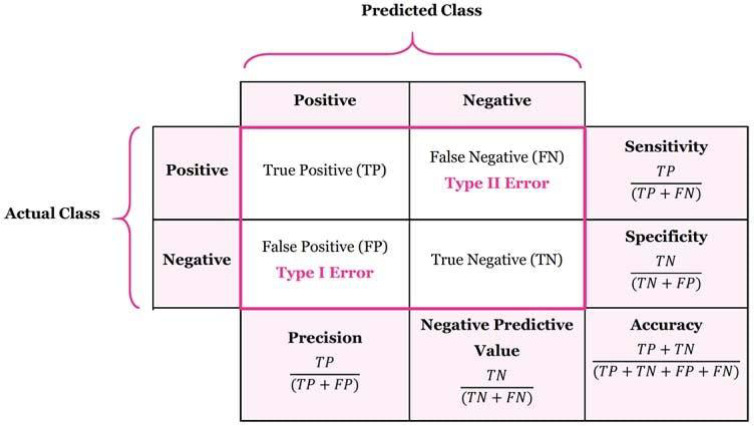
A confusion matrix with mathematical equations representing the performance metrics.

A detailed schematic of the confusion matrix, along with the mathematical equations used to derive performance measures, is presented in Fig. 6.

One of the most commonly utilized performance metrics in artificial intelligence is the Area Under the Receiver Operating Characteristic Curve (AUC-ROC). The AUC provides a scalar value that quantifies the discriminative capability of a classification model by measuring the area under the ROC curve, which is generated by plotting the true positive rate (sensitivity) against the false positive rate ($\:\frac{FP}{FP+TN}$). These values are derived directly from the confusion matrix. An AUC value approaching 1.0 reflects excellent class separation capability, while a value near 0.5 indicates that the model performs at chance level, offering no useful discriminative power. Due to its threshold-independent nature and robustness against class imbalance, AUC has become a standard metric for evaluating the effectiveness of binary and multiclass classifiers in AI applications.

Confidence intervals (CIs) play a critical role in the statistical evaluation of AI models, particularly in ML and DL, by quantifying the variability and uncertainty associated with performance estimates derived from finite data samples. A CI provides a probabilistic range within which the true value of a given performance metric—such as accuracy, precision, or AUC—is expected to reside, typically at a 95% confidence level. This estimation is particularly important in cross-validation or limited-sample scenarios, where point estimates alone may be misleading. The width of the CI reflects the stability and generalizability of the model: narrower intervals indicate more reliable and consistent performance, while wider intervals suggest greater variability. As such, CIs are increasingly employed in recent literature to complement average metric scores and provide a more rigorous, statistically grounded interpretation of model robustness. The formal equation used for CI computation is provided in Eq. 4.

4 $$CI = ~\bar{x} \pm z~x~\frac{s}{{\sqrt n }}~$$

As shown in Eq. (4), *x̄* denotes the sample mean (e.g., the average accuracy obtained from 10-fold cross-validation), *z* represents the z-score corresponding to the selected confidence level (typically 1.96 for a 95% confidence level), *s* is the sample standard deviation, and *n* denotes the sample size (e.g., the number of folds). In this study, since the Stress-Lysis dataset does not provide any predefined partitioning ratios, both specific train-test split ratios and 10-fold cross-validation with 95% Cis were employed to evaluate the model’s performance. The resulting outcomes were subsequently analyzed and reported.

### Performance comparison of the proposed ligthweight St-CNN architecture and ML methods on the Stress-Lysis dataset

**Fig. 7:**
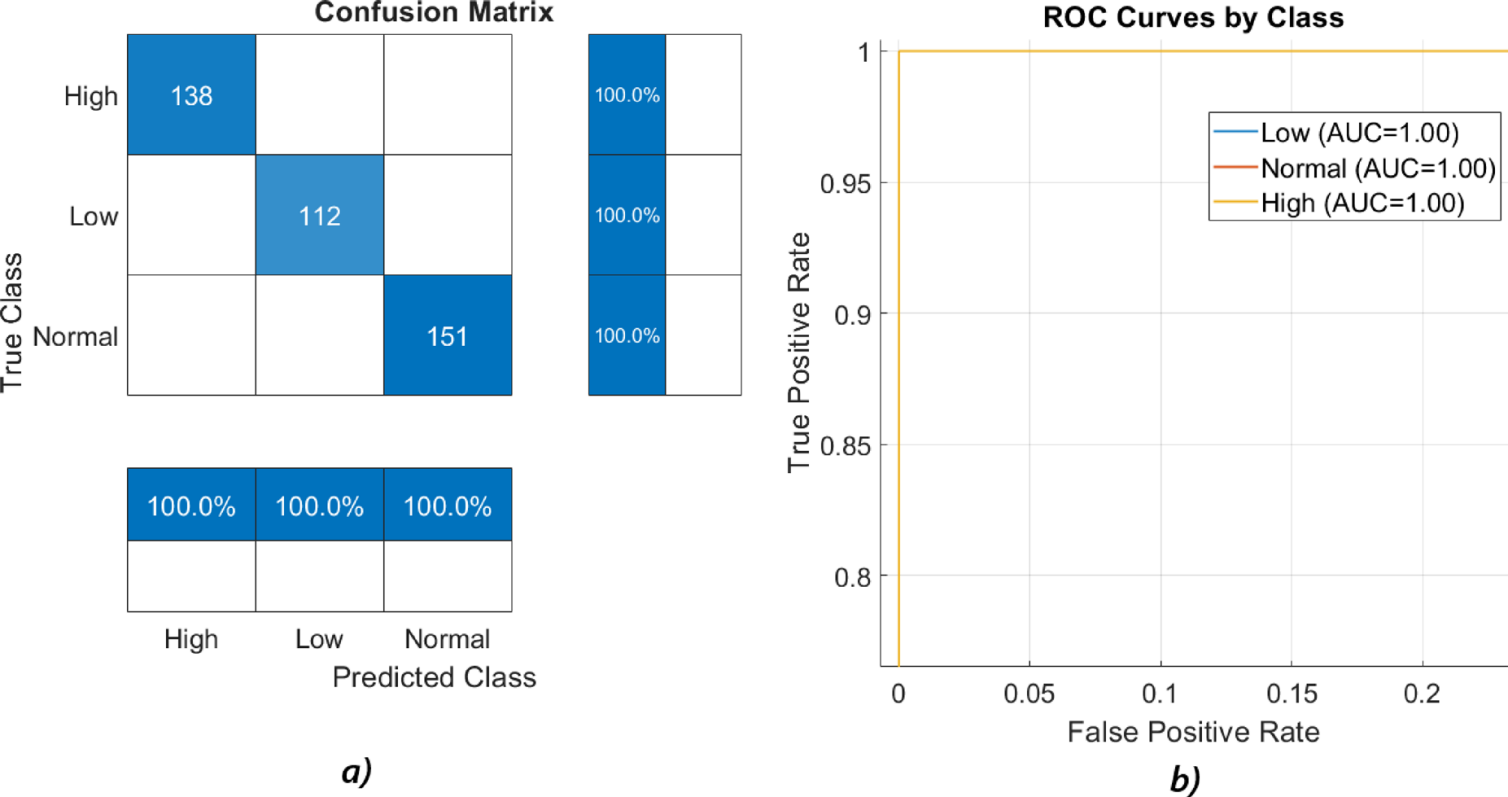
Results of the performance evaluation of the proposed method: (**a**) Confusion matrix, (**b**) ROC curves by class.

In this study, the proposed St-CNN architecture was trained according to the procedures detailed in Sect. 4.2 and subsequently evaluated on the designated test subset of the dataset. After the evaluation, a confusion matrix was constructed to quantify the performance of the proposed model, with accuracy serving as the principal performance metric. To facilitate a comprehensive performance comparison, four additional training sessions were conducted utilizing various ML algorithms: KNN with k = 3 and k = 5, DT, and SVM (with linear as a kernel function). Each of these ML methods was applied to the same dataset following an identical protocol as used for the St-CNN model. After training, each ML model was evaluated on the test subset, and corresponding confusion matrices were generated to assess their performance, with both accuracy and F1 scores calculated as key performance metrics. The comparative accuracy and F1 score results for the proposed St-CNN model and the traditional ML methods are summarized in Table 2, while the confusion matrix and ROC curve of the proposed St-CNN model are illustrated in Fig. 7.

**Table 2:** Performance comparison with accuracy metrics of the proposed St-CNN architecture and ML methods.

Method	Method	Accuracy (%)	F1 Score (%)
Machine Learning	KNN (k = 3)	99.75	99.74
Machine Learning	KNN (k = 5)	99.5	99.48
Machine Learning	SVM	99.75	99.77
Machine Learning	DT	99.75	99.77
**Deep Learning**	**St-CNN**	100	100

Table 2 illustrates the comparative performance of the proposed St-CNN architecture against various ML methods. The results indicate that the proposed St-CNN architecture significantly outperforms the ML methods, achieving the highest accuracy with exemplary performance. Among the ML methods evaluated, DT, KNN with k = 3, and SVM demonstrated performance levels closest to those of the proposed St-CNN model. Notably, all ML methods, with the exception of KNN with k = 5, exhibited similar performance metrics. The DL-based St-CNN method thus demonstrates superior performance in terms of both accuracy and operational efficiency when compared to traditional ML methods. Additionally, as illustrated in Fig. 7(b), the ROC curves for all three classes (Low, Normal, High) achieved an AUC of 100%, confirming the model’s outstanding ability to distinguish between classes with perfect sensitivity and specificity.

### Investigation of the optimal dataset partition ratio for the Stress-Lysis dataset using the proposed method

The Stress-Lysis dataset does not come pre-divided into training and testing subsets, which is crucial for conducting standardized performance evaluations of classification algorithms. Therefore, this study explored various data splitting ratios to identify the most effective partitioning strategy. The models were trained using the hyperparameters specified in Table 1. Following the training phase, the St-CNN model was evaluated on test subsets corresponding to each data splitting ratio. For each ratio, a confusion matrix was generated to assess performance, and accuracy metrics were computed. The results of the accuracy comparisons across different data splitting ratios are summarized in Table 3. To evaluate the statistical reliability of the proposed model under varying data conditions, experiments were conducted using four different training–test split ratios: 60–40, 70–30, 80–20, and 90–10. For each split configuration, the model was trained and tested ten times with different random seeds to mitigate the impact of initialization and data shuffling on performance. This repeated experimentation allowed for the computation of average accuracy values across multiple independent trials, providing a more robust and generalizable measure of model performance. Furthermore, a one-way ANOVA test was performed to determine whether the observed differences in mean accuracy among the split ratios were statistically significant. The corresponding mean accuracies and ANOVA results are presented in Table 3.

**Table 3:** Comparison of the performance of the proposed method on the Stress-Lysis dataset across various data splitting ratios.

Stress-Lysis	Train	Test	Accuray	Sensitivity	Specificity	Precision	F1 score	Mean Accuracy
**Split Ratio**	60	40	99.63	99.70	99.83	99.51	99.60	99.79
	70	30	99.83	99.86	99.93	99.79	99.82	99.78
	80	20	**100**	**100**	**100**	**100**	**100**	**99.88**
	90	10	**100**	**100**	**100**	**100**	**100**	99.75

*p-value (ANOVA): 0.7936*

* Performance metrics are shown as percentages and the highest performance is bolded in each column.

As presented in Table 3, the proposed method exhibited consistently high classification performance across all training–testing split configurations on the Stress-Lysis dataset. Notably, the 80–20 and 90–10 configurations achieved perfect scores across all evaluation metrics—including accuracy, sensitivity, specificity, precision, and F1 score—while the 60–40 and 70–30 partitions yielded comparably strong results. Based on the average accuracies computed from ten independent experimental runs for each split ratio, the 80–20 configuration attained the highest mean accuracy at 99.88%. To determine whether the observed performance differences across data partitioning schemes were statistically significant, a one-way analysis of variance (ANOVA) was conducted on the mean accuracy scores. The analysis yielded a p-value of 0.7936, indicating that the performance variations among the different training–testing ratios were not statistically significant. Rather than suggesting a limitation, this result underscores the model’s robustness and invariance to data partitioning, demonstrating its ability to maintain high performance across a range of experimental conditions. The absence of statistically significant differences, coupled with uniformly high classification accuracy, highlights the method’s strong generalization capacity. Furthermore, the closely aligned convergence patterns observed in both the training and validation loss curves (Fig. 5) reinforce this conclusion, indicating that the model remains stable and free from overfitting across all tested configurations. Based on the comprehensive results obtained from all experimental tests and performance evaluations, the 80–20 training–testing split configuration yielded the most favorable overall performance of the proposed method on the Stress-Lysis dataset.

### Performance evaluation of the proposed method on the Stress-Lysis dataset using 10-fold cross-validation

In this study, the 10-fold cross-validation technique was also used for a detailed performance evaluation of the Stress-Lysis dataset, and the results are shown in Table 4.

**Table 4:** 10-fold cross validation performance results on the Stress-Lysis dataset with the proposed method.

Fold	Accuracy	Precision	Sensitivity	Specificity	F1 Score	AUC
1	100	100	100	100	100	100
2	100	100	100	100	100	100
3	100	100	100	100	100	100
4	100	100	100	100	100	100
5	100	100	100	100	100	100
6	100	100	100	100	100	100
7	99.50	99.58	99.53	99.72	99.55	100
8	100	100	100	100	100	100
9	99.50	99.35	99.58	99.78	99.46	100
10	99.50	99.35	99.58	99.78	99.46	100
Overall	99.85	99.83	99.87	99.93	99.85	100

According to the cross-validation results presented in Table 4, the proposed St-CNN architecture achieves superior performance in the AUC metric across all folds, while the other metrics consistently reach near-perfect accuracy levels above 99%. From another perspective, to estimate the overall performance of the model on the dataset, the overall values of all performance metrics were calculated. However, relying solely on overall values from cross-validation may be misleading when evaluating model performance. Therefore, 95% CIs were computed to assess the reliability and stability of the obtained performance metrics. The CI values for each performance metric are presented in Table 5.

**Table 5:** Confidence intervals (95%) of cross-validation performance results.

Metric	Mean	CI (with 95%)	CI Error
Accuracy	0.9985	[0.9970, 1.0000]	0.0015
Precision	0.9983	[0.9965, 1.0000]	0.0018
Recall	0.9987	[0.9974, 1.0000]	0.0013
Specificity	0.9993	[0.9986, 1.0000]	0.0007
F1 Score	0.9985	[0.9969, 1.0000]	0.0015
AUC	1.0000	[1.0000, 1.0000]	0.0000

As shown in Table 5, the upper bounds of the CIs for all performance metrics reach full accuracy, indicating minimal variation across cross-validation folds. This demonstrates that the proposed St-CNN architecture achieves consistently high performance not only across different dataset partitioning ratios but also under cross-validation-based evaluation.

### Ablation study with the proposed method

Considering the FC layers together with the normalization and ReLU activation layers as a dense block, an ablation study was conducted to investigate the effect of varying the number of such blocks on the performance and training time of the proposed St-CNN architecture. The results obtained from this study are presented in Table 6.

**Table 6:** Results of ablation study with the proposed method using various numbers of FC layers.

Method	FC Layer	Accuracy	Training Time
St-CNN (Proposed Method)	2	100	01:16s
	3	99.50	01:53s
	4	99.50	01:26s
	5	99	01:20s
	6	98.75	01:35s

As shown in Table 6, the model with two FC layers achieved the highest classification accuracy of 100% with the shortest training time of 1 min and 16 s. Increasing the number of FC layers beyond two resulted in a slight decrease in accuracy, accompanied by fluctuations in training time. Architectures with three or four FC layers maintained a relatively high accuracy of 99.50% but exhibited longer training times. Configurations with five and six FC layers further reduced performance to 99% and 98.75%, respectively. These findings suggest that a lightweight two-layer structure is the optimal configuration for the given task and dataset, as it not only optimizes classification performance but also enhances computational efficiency. In conclusion, the findings of the ablation study demonstrate that the proposed method achieves effective accuracy and training efficiency and represents a suitable solution for stress detection tasks in edge computing environments.

## Discussion

This section contextualises the proposed method by discussing the literature related to AI-based stress detection examined in Sect. 2. In this study, a comprehensive performance evaluation of the proposed St-CNN architecture was performed using ablation studies, cross-validation, and various data splitting ratios. Since there is no specific training/test ratio in the Stress-Lysis dataset, comparing the cross-validation performance of the proposed method (based on 95% CI) with the literature provides a more appropriate and reliable comparison. Accordingly, a comparison of the structural and computational characteristics that determine the basic performance metrics, system complexity, and suitability of the method for edge devices between the proposed method and previous studies is presented in Table 7.

**Table 7:** A performance comparison of the proposed method with the-state-of-art methods on the Stress-Lysis dataset.

Source	Method	Accuracy (%)	Parameter (count)	FLOPs (per inference)	System Complexity
Rachakonda et al. (2019)^45^	DNN	99.7	503	963	Less Complex
Al-Atawi et al. (2023)^38^	Ensemble Learning	99.5	Unreported (High est.)	Unreported (High est.)	Moderate to High
Mohod et al. (2024)^46^	SVM	99.0	Unreported (~ 200+) est.	Unreported (Mid est.)	Moderate
Suraj Arya and Ramli (2024)^47^	Naive Bayes	90.0	Unreported (Low est.)	Unreported (Low est.)	Moderate
Bobade and Vani (2020)^48^	ANN	95.21	Unreported (~ 300–800) est.	Unreported (Mid est.)	Moderate
Kadu et al. (2024)^49^	Logistic Regression	99.8	Very Low (Arduino-based)	Low	Less Complex
Mukherjee & Roy (2024)^50^	CNN-TLSTM	99.72	~ 1.39 M	High	High
**Proposed Method** **(Cross Validation)**	**Lightweight St-CNN**	**0.9985** **(95% CI: [0.9970**,** 1.0000])**	**115 (Very Low)**	**287 (Low)**	**Less Complex**

*Best performance value is shown in bold style. Also, “est.” term is an abbreviation for “estimated”.

As shown in Table 7, the proposed lightweight St-CNN model achieves a favorable trade-off between classification performance and computational efficiency when compared to existing approaches evaluated on the Stress-Lysis dataset. Although several prior methods, such as the DNN proposed by Rachakonda et al.^45^ and the ensemble model by Al-Atawi et al.^38^, report high levels of accuracy (99.7% and 99.5%, respectively), these models involve considerably higher computational costs. In contrast, the proposed St-CNN achieves excellent accuracy (100%) with minimal computational requirements, significantly reducing resource demands compared to other state-of-the-art approaches. In addition, traditional ML methods, including SVM, ANN, and Naive Bayes, generally exhibit lower predictive performance and do not offer the same efficiency or scalability for edge deployment. These findings highlight the practical suitability of the proposed model for stress detection tasks in real-time and resource-constrained environments.

Beyond its high classification performance, the proposed St-CNN model stands out for its architectural simplicity and computational efficiency, as detailed in Table 7. While previous DL-based models, such as the DNN architecture by Rachakonda et al.^45^, also report high accuracy, they rely on more complex structures with a higher number of parameters and computational cost. In contrast, the St-CNN achieves perfect accuracy while maintaining a low parameter count and FLOPs, thereby substantially reducing computational overhead. Similarly, although ensemble-based approaches like that of Al-Atawi et al.^38^ achieve comparable accuracy, their reliance on multiple classifiers leads to increased complexity and reduced suitability for real-time deployment on edge devices. Classical ML methods such as SVM and Naive Bayes, while computationally lighter, fall short in predictive performance and scalability. What differentiates the proposed St-CNN from these prior approaches is its ability to combine the learning capacity of deep models with a compact structure designed for low-latency environments. By maintaining both accuracy and efficiency, the St-CNN presents a compelling alternative for real-world stress detection systems, particularly in edge-based and wearable technologies where computational resources are limited. In addition to its architectural and computational contributions, this study has also addressed the challenge posed by the absence of predefined training and testing splits in the Stress-Lysis dataset. To identify the most effective data partitioning strategy, multiple training/testing ratios have been evaluated, and the configuration that yielded the highest classification performance has been selected for implementing the proposed method. These experimental results have been presented in detail, demonstrating that the model is adaptable to varying data distributions. Furthermore, a comprehensive cross-validation analysis has been conducted to ensure a more rigorous assessment of the model’s generalization capability. The inclusion of CIs has further demonstrated the stability and reliability of the obtained performance metrics. In addition, to evaluate the lightweight design and performance of the proposed method, an ablation study was conducted based on dense blocks containing FC layers.

As shown in Tables 3, 4, 5 and 6, and 7, comprehensive performance evaluations and comparisons, including CIs, cross-validation, and ablation studies, were conducted to validate the effectiveness of the proposed model. According to these results, the model demonstrated exceptional and robust performance even at lower design complexities with the selected hyperparameters. The results of this study also show that the proposed St-CNN architecture achieves the highest performance compared to previous studies in both cross-validation and dataset splitting ratio evaluation, even with the lightest configuration.

As observed in previous studies, the Stress-Lysis dataset has generally yielded high performance levels across various AI-based stress detection approaches. However, to the best of our knowledge, no prior work has achieved performance close to perfection while also incorporating additional evaluation strategies such as cross-validation. In this study, the proposed St-CNN architecture achieved an accuracy of 99.85% (95% CI: [99.70%, 100.00%]) despite the inherent class imbalance in the dataset. As illustrated in Fig. 5, no indications of overfitting were observed, and this is further supported by the consistently high performance obtained across all evaluation metrics. A comprehensive assessment, including ablation studies, cross-validation, and evaluations with varying data splitting ratios, was conducted. Potential concerns regarding limited real-world applicability and the cost of misclassifications were addressed by employing multiple complementary performance metrics (precision, recall, specificity, F1-score, and AUC) and confirming model stability through narrow confidence intervals across folds. Consequently, the proposed approach surpasses previously reported results in both accuracy and robustness, establishing a new benchmark for Stress-Lysis-based stress detection methods.

From an application standpoint, real-time and wearable IoMT systems are typically constrained by limited processing power and memory resources. To address these constraints, a lightweight DL model—St-CNN—has been introduced in this study, specifically designed to achieve robust classification performance with minimal computational overhead. The model’s streamlined architecture—characterized by a notably low number of trainable parameters and reduced FLOPs—has been shown to significantly enhance its feasibility for edge deployment. By leveraging physiological data acquired through wearable sensors, the proposed method has enabled continuous stress monitoring, real-time feedback, and proactive intervention. In summary, with its effective performance and compact design, the St-CNN architecture has demonstrated strong potential as a foundational component of AI-driven health monitoring systems, particularly in edge computing scenarios where low latency, energy efficiency, and on-device inference are essential.

###  Limitations

One key limitation of this study is the relatively small size of the Stress-Lysis dataset. Despite achieving high classification accuracy, there is a potential risk of overfitting due to the limited amount of training data. Although several regularization techniques were applied, further testing with larger and more diverse datasets is necessary to confirm the model’s generalizability. Another limitation pertains to the limited variety of stress-inducing activities and environmental conditions represented in the Stress-Lysis dataset. Stress is influenced by a wide range of external and personal factors, and the current dataset may not comprehensively capture these variations. Future research should consider incorporating diverse stress scenarios and sensor configurations to improve the model’s applicability. Furthermore, to better understand and characterize the inherent challenges posed by such datasets, future studies should also explore data complexity-based evaluations. These approaches^59– 60^—by analyzing feature overlap, class separability, and intrinsic dimensionality—can offer more informed guidance in selecting or designing suitable models for physiological signal-based stress detection tasks.

###  Ethical considerations with edge computing AI for stress detection

Computer-aided systems designed for medical applications, such as stress detection, often involve the processing of physiological data obtained from individuals, which introduces certain ethical responsibilities. To address these concerns, recent approaches have shifted towards the use of edge computing, which processes data locally on devices rather than in cloud servers, thereby minimizing the risks of data breaches and unauthorized access in medical contexts^47– 48^. Nevertheless, IoT devices and sensors employed in edge computing often lack the advanced hardware capabilities of next-generation computing systems, which can limit the implementation or support of AI technologies due to hardware constraints. The limited processing capacity of these devices poses challenges for integrating complex AI architectures. Consequently, the utilization of edge computing becomes critical in medical applications where ethical considerations, such as stress detection, are paramount, and where the need for accurate, real-time data processing using AI methods is indispensable. In this context, the development of lightweight and highly accurate AI models is essential to facilitate their integration into edge computing frameworks.

In this study, a lightweight and high-performance St-CNN architecture is proposed, demonstrating superior performance relative to the existing literature. The proposed architecture, with its demonstrated capabilities, emerges as a promising tool for privacy-preserving edge computing AI approaches, which can enhance proactive stress management. Such an approach not only bolsters user trust in an era characterized by the widespread adoption of wearable devices and personal health applications but also offers effective and reliable solutions for stress detection applications. At this point, in this study, St-CNN architecture has been developed to serve edge computing by achieving the highest performance in the literature with various performance tests for stress detection.

## Conclusion

This paper proposes a lightweight St-CNN architecture for detecting stress levels based on human activity data and rigorously evaluates its performance using the widely recognized Stress-Lysis dataset. The results demonstrate that the St-CNN significantly outperforms traditional ML methods such as KNN, SVM, and DT, achieving an exceptional accuracy of 100%. A comparative analysis with state-of-the-art techniques further underscores the effectiveness of the proposed approach and positions it among the leading methodologies in the field, particularly for deployment on edge devices. Additionally, the identification of an optimal data partitioning ratio contributed to maximizing the model’s performance. Cross-validation conducted on the Stress-Lysis dataset further validated the robustness and generalizability of the proposed method. Overall, the findings highlight the potential of St-CNN as a vital component in real-time, edge-computing-based AI systems for stress monitoring and management using wearable IoMT devices. Moreover, the comparison with previous studies indicates that the proposed method ranks among state-of-the-art approaches, offering both high performance and lower computational cost, along with reduced system complexity compared to existing methods. Future work will explore more cost-efficient St-CNN variants and assess their performance on larger stress datasets real-world environments, leveraging advanced learning strategies to further enhance accuracy and generalizability.

## Data Availability

The Stress-Lysis dataset used in this study is publicly available and can be accessed at https://www.kaggle.com/datasets/laavanya/stress-level-detection.
